# Mutation in SLC6A9 encoding a glycine transporter causes a novel form of non-ketotic hyperglycinemia in humans

**DOI:** 10.1007/s00439-016-1719-x

**Published:** 2016-08-01

**Authors:** Majid Alfadhel, Marwan Nashabat, Hanan Al Qahtani, Ahmed Alfares, Fuad Al Mutairi, Hesham Al Shaalan, Ganka V. Douglas, Klaas Wierenga, Jane Juusola, Muhammad Talal Alrifai, Stefan T. Arold, Fowzan Alkuraya, Qais Abu Ali

**Affiliations:** 1 Division of Genetics, Department of Pediatrics, King Abdullah International Medical Research Centre, King Saud bin Abdulaziz University for Health Sciences, Ministry of National Guard-Health Affairs (NGHA), King Abdulaziz Medical City, PO Box 22490, Riyadh, 11426 Saudi Arabia; 2 Medical Imaging Department, King Abdullah International Medical Research Centre, King Saud bin Abdulaziz University for Health Sciences, Ministry of National Guard-Health Affairs (NGHA), King Abdulaziz Medical City, Riyadh, Saudi Arabia; 3 Department of Pediatrics, College of Medicine, Qassim University, Almulyda, Saudi Arabia; 4 GeneDx, Gaithersburg, MD 20877 USA; 5 Department of Pediatrics, Section of Genetics, The University of Oklahoma Health Sciences Center (OUHSC), Oklahoma City, OK USA; 6 Neurology Division, Department of Pediatrics, King Abdullah International Medical Research Centre, King Saud bin Abdulaziz University for Health Sciences, Ministry of National Guard-Health Affairs (NGHA), King Abdulaziz Medical City, Riyadh, Saudi Arabia; 7 Division of Biological and Environmental Sciences and Engineering (BESE), Computational Bioscience Research Center (CBRC), King Abdullah University of Science and Technology (KAUST), Thuwal, 23955-6900 Saudi Arabia; 8 Department of Genetics, King Faisal Specialist Hospital and Research Center, Riyadh, Saudi Arabia; 9 Department of Anatomy and Cell Biology, College of Medicine, Alfaisal University, Riyadh, Saudi Arabia

## Abstract

Glycine cleavage system (GCS) catalyzes the degradation of glycine and disruption of its components encoded by *GLDC*, *AMT* and *GCSH* are the only known causes of glycine encephalopathy, also known as non-ketotic hyperglycinemia (NKH). In this report, we describe a consanguineous family with one child who presented with NKH, but harbored no pathogenic variants in any of the three genes linked to this condition. Whole-exome sequencing revealed a novel homozygous missense variant in exon 9 of *SLC6A9* NM_201649.3: c.1219 A>G (p.Ser407Gly) that segregates with the disease within the family. This variant replaces the highly conserved S407 in the ion-binding site of this glycine transporter and is predicted to disrupt its function. In murine model, knockout of *Slc6a9* is associated with equivalent phenotype of NKH, namely respiratory distress and hypotonia. This is the first demonstration that mutation of the glycine transporter can be associated with NKH in humans.

## Introduction

Although glycine is the smallest amino acid, it is considered a conditionally essential amino acid, as de novo synthesis may not be sufficient (Wang et al. 2013). It is essential for various processes in the body, including protein, glutathione, creatine, purine, heme, and serine synthesis (Dai et al. 2013; Hall 1998). It is also essential for bile acid conjugation (Hafkenscheid and Hectors 1975), as well as playing an important role in immune modulation through the glycine-gated chloride channels (Zhong et al. 2003). Moreover, glycine acts as an inhibitory neurotransmitter in central nervous system (CNS) by hyperpolarizing the post-synaptic glycinergic neurons (Legendre 2001). Glycine levels are controlled by two main mechanisms; first, glycine degradation, mainly by the Glycine Cleavage System (GCS) and to a lesser extent formation of serine (Wang et al. 2013), and second, by glycine transport system which is mediated by two sodium-dependent carriers, mainly GLYT1 and GLYT2. Glycine Transporter 1 (GlyT1) balances the extracellular glycine concentration predominantly in CNS (Betz et al. 2006).

Disruption of the GCS leads to glycine encephalopathy or non-ketotic hyperglycinemia (NKH), where CSF-to-plasma glycine ratio is elevated. In utero, fetal hiccups and myoclonus might be noticed, which may persist after delivery in addition to hypotonia and respiratory disturbances. Later, in life, patients develop seizures and intellectual disability (Dalla Bernardina et al. 1979). Nearly all cases of NKH have been found to harbor disease-causing autosomal recessive variants in the genes encoding the different components of the GCS: glycine decarboxylase (*GLDC*), which encodes P protein, aminomethyltransferase gene (*AMT*), which encodes T protein; and glycine cleavage system H gene (*GCSH*), which encodes H protein (Applegarth and Toone 2006).

Glycine encephalopathy caused by glycine transporter system was proposed as a distinct entity only once in an atypical NKH case. Mayor et al. (1984) described a patient who presented with classic symptoms of NKH; however, no confirmatory mutation analysis was conducted (Mayor et al. 1984). In this study, we suggest that NKH can, indeed, be caused by mutation of the glycine transporter, which we suggest is a novel, albeit rare cause of NKH in humans.

## Materials and methods

### Human subjects

The index case underwent full clinical evaluation by a clinical geneticist for her multisystem disorder (see below). Standard clinical-exome consent was obtained from the proband’s parents. Blood samples were collected from index case and parents in EDTA tubes for DNA extraction, and trio whole-exome sequencing analysis (proband and both parents) was performed at a CLIA-certified clinical diagnostic laboratory.

### Exome analysis

Genomic DNA was extracted from whole blood for the affected proband and family members (both unaffected parents and unaffected sister). Exome sequencing at GeneDx was performed on exon targets isolated by capture using the Agilent Clinical Research Exome kit (Agilent Technologies, Santa Clara, CA). The sequencing methodology and variant interpretation protocol have been previously described (Tanaka et al. 2015). The general assertion criteria for variant classification are publicly available on the GeneDx ClinVar submission page (http://www.ncbi.nlm.nih.gov/clinvar/submitters/26957/).

Percent covered at 10× for the proband was 96.37, while that for mother and father was 96.22 and 96.42, respectively. Mean coverage for the proband was 132.67, while that for mother and father was 116.72 and 130.07, respectively. Sanger sequencing of *AMT*, *GLDC*, and *GCSH,* which are mutated in NKH, was performed to further rule out mutation of any of these genes as the cause of the patient’s phenotype.

### Computational structural analysis of mutants

Sequences were retrieved from the Uniprot database. BLAST and SwissModel (Arnold et al. 2006) were used to search for suitable structural templates in the Protein Data Bank (PDB). SwissModel was used to produce homology models. Models were manually inspected and evaluated using the Pymol program (pymol.org).

## Results

### Clinical report

**Fig. 1:**
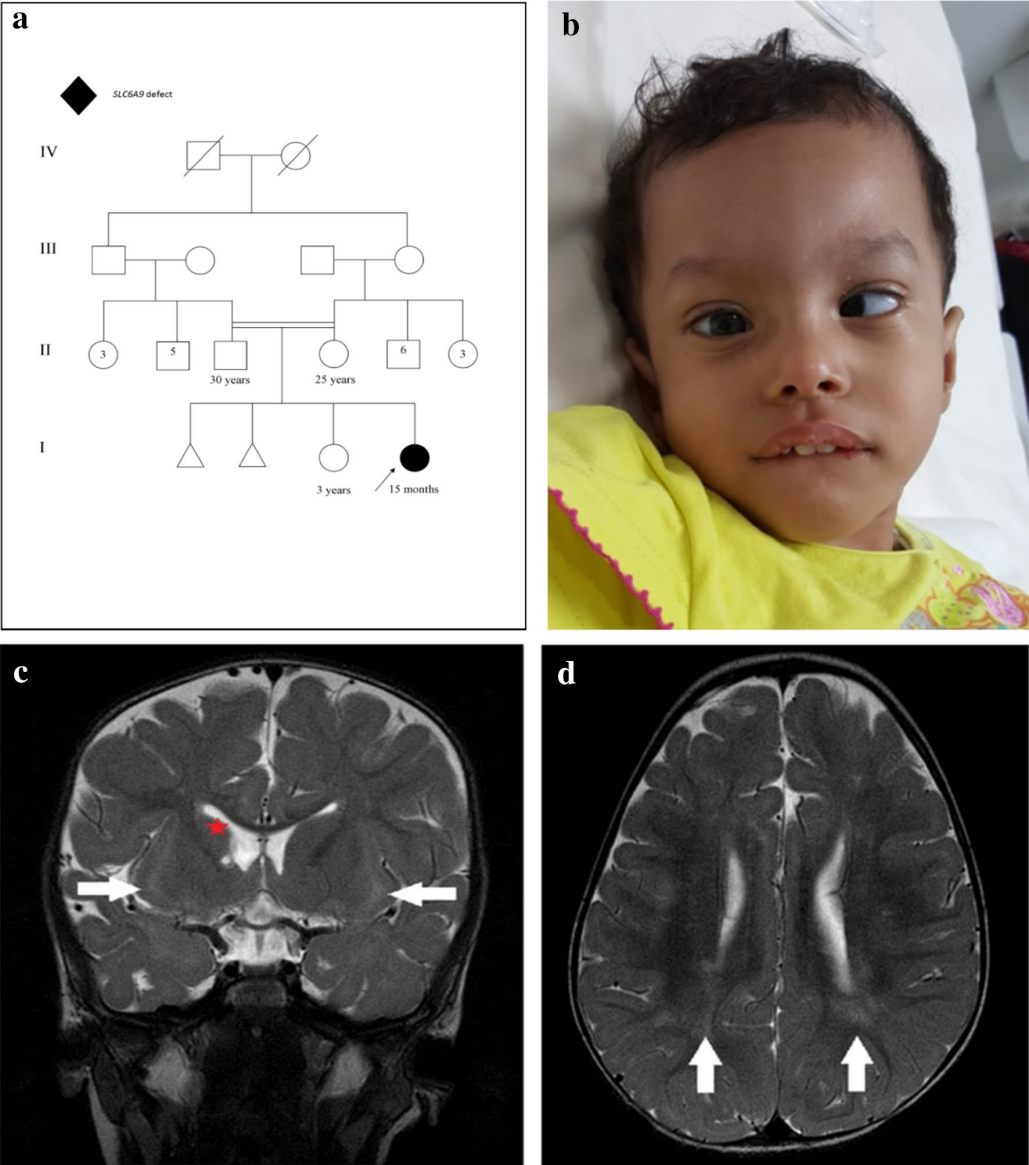
**a** Family pedigree, **b** esotropia and microcephaly, broad forehead, esotropia, low set ears, retrognathia, deep prominent philtrum and sparse eyebrows, **c** selected MR image of the brain at the level of basal ganglia, coronal T2-WI shows bilateral subinsular white matter hyper intensity (*arrows*) and right head of caudate atrophy with a tiny cyst (*star*), **d** axial T2-WI shows bilateral scattered subcortical and periventricular white matter hyperintensities (*arrows*)

The index case is a 15-month-old girl product of full-term pregnancy via normal spontaneous vaginal delivery, born to first cousin Saudi parents (Fig. 1). Pregnancy was complicated by polyhydramnios, and there was no history of abnormal fetal movements. Maternal obstetric history was significant for two first trimester miscarriages and a healthy older daughter (G4P2 + 2) with the same partner. Birth growth parameters include weight 2900 g (−0.8 SD), length 50 cm (0.45 SD), and OFC 34.5 cm (0.52 SD). Shortly, after birth, she developed shallow breathing and acute respiratory distress that required intubation and ventilatory support for 1 month prior to successful extubation in the NICU. Neonatal course, starting at age of 3 days, was further complicated by seizure-like episodes, which were medically controlled with phenobarbital and phenytoin. Subsequently, it was noted that these symptoms were likely exaggerated startle reflexes provoked by sudden loud sounds and tactile stimulation, i.e., hyperekplexia. All antiepileptic medications were gradually tapered, as such seizure-like episodes and startle reflexes improved over time and completely subsided by age 6 months. In addition, the patient had hypotonia, weak cry, difficulty swallowing which lead to aspiration, multiple hospitalizations, and eventually insertion of gastrostomy tube. Clinical follow-up at 15 months of age showed failure to thrive and microcephaly: weight was 6.8 kg (−2.8 SD), length 73 cm (−1.6 SD), and head circumference 40.5 cm (−3.7 SD). Subtle facial dysmorphic features were noted: microcephaly, broad forehead, esotropia, low set ears, retrognathia, deep prominent philtrum, and sparse eyebrows (Fig. 1). Developmentally, she had global delay, including motor and speech. Neurological examination showed central hypotonia with brisk deep tendon reflexes peripherally; musculoskeletal examination showed bilateral joint laxity around the elbows with bilateral club feet. Rest of clinical exam was unremarkable. The following laboratory investigations were unremarkable: newborn screening, CBC, renal function, liver function, ammonia, serum lactic acid, urine organic acids, chromosomal analysis, CGH microarray, and clinical sequencing of *AMT*, *GLDC*, and *GCSH* genes.

Plasma amino acids showed mild elevation of glycine at 288 µmol/L (normal: 73–241). Urine amino acids also showed mild elevation of glycine at 393 µM/mM Cr (normal: 110–356), methionine at 66 µM/mM Cr (normal: 7–29), ethanolamine at 98 µM/mM Cr (normal: not detected), and ornithine at 14 µM/mM Cr (normal: <8). This prompted CSF amino acid analysis, which revealed elevated glycine at 21 µmol/L (normal: 2.2–14.2) and CSF/plasma glycine ratio, was elevated at 0.07(normal: <0.02). Electroencephalogram (EEG) and echocardiogram were both normal. Skeletal survey showed right developmental dysplasia of the hip (DDH), superior dislocation of the right proximal femur, and bilateral club feet. Magnetic resonance imaging (MRI) of the brain showed bilateral subinsular white matter T2 hyperintensity, right head of caudate atrophy with tiny cyst, and bilateral scattered subcortical and periventricular white matter changes (Fig. 1).

Whole-exome sequencing showed a novel homozygous missense variant in exon 9 of the *SLC6A9* gene: NM_201649.3: c.1219 A>G (p.Ser407Gly), as both parents and the older sister were heterozygous for this variant. This missense variant has not previously been reported in any of the publicly available population catalogs (1000 Genomes, NHLBI ESP/EVS, ExAC), >1400 individuals of Arabic background in GeneDx internal exome database, or 2000 ethnically matched (Saudi) exomes from the Saudi Human Genome Program database. Moreover, the discovered variant resides within the autozygome. This missense change is predicted to be pathogenic by various in silico models: SIFT (0.003), PolyPhen (1) and CADD (26.9).

**Fig. 2:**
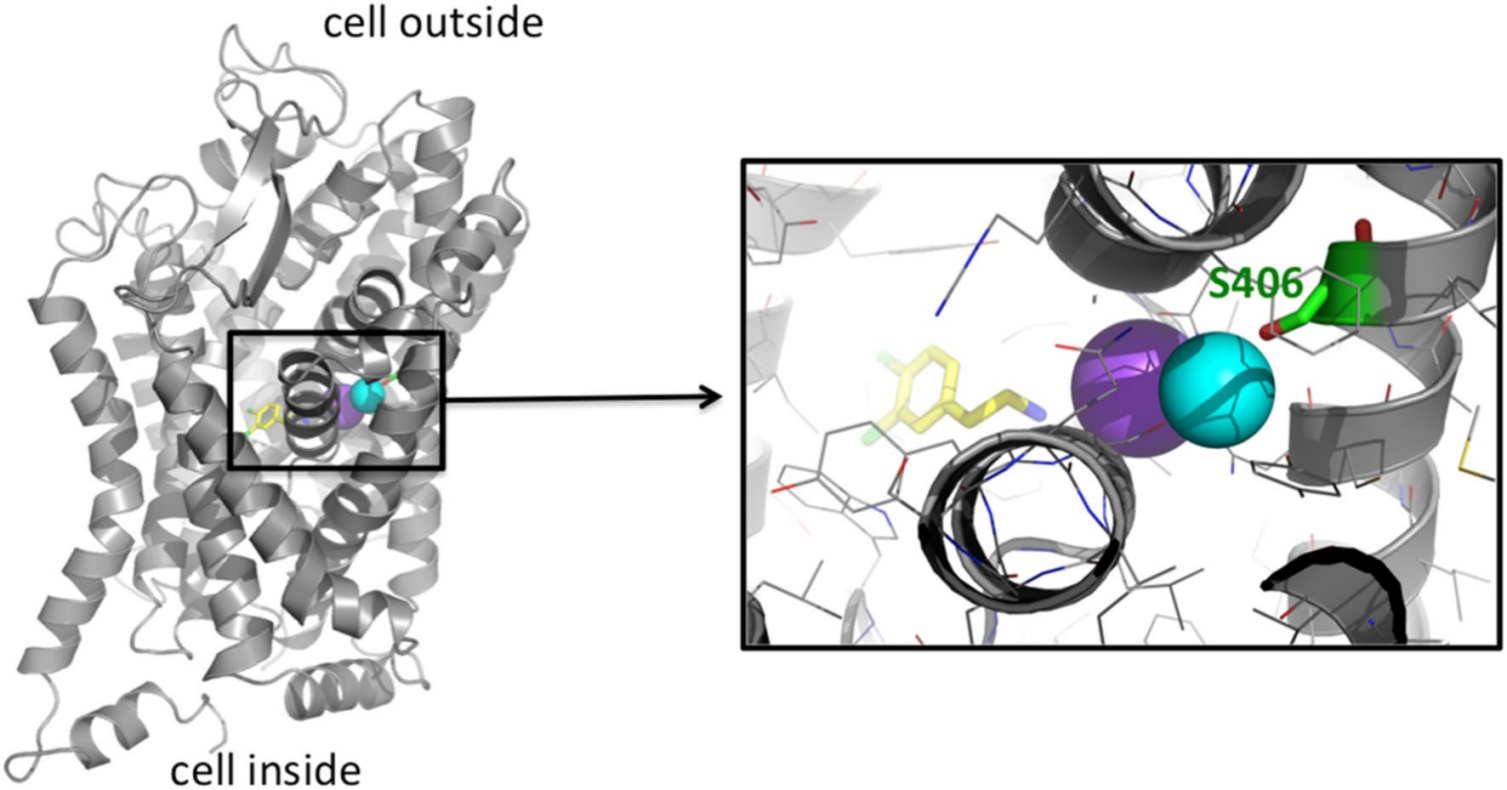
3D homology model of human SLC6A9, based on the dopamine transporter (PDB 4xpa). The dopamine analogue 3,4-dichlorophenethylamine (*yellow*), sodium (*magenta*), and chloride (*cyan*) ions have been taken over from the dopamine receptor structure that served as template. The bound ions are expected to be located in the wild-type (but not the S406 mutant) SLC6A9, as they are in the dopamine transporter, because of strict conservation of the ion-binding site. However, the dopamine analogue is of course replaced by glycine in SLC6A9, and the dopamine analogue is only displayed to illustrate the location of S406 with respect to the ligand molecule

The SLC6A9 protein can be modelled based on the crystal structure of the 46 % identical dopamine transporter (PDB accession 4xpa and related entries) from Drosophila, or the 43 % identical human sodium-dependent serotonin transporter (PDB 5i6z). In both these template sequences and structures, the serine at position 407 is conserved. Ser407 is located in the middle of transmembrane helix 7. Although it is not in direct contact with the ligand channel, and positioned in a distance of about 7–10 Å to the ligand, Ser407 contributes to a cavity that binds a chloride and sodium ion in the highly similar dopamine transporter (Wang et al. 2015) (Fig. 2). This ion-binding site allows the receptor to correlate substrate transport with pre-existing chloride and sodium gradients. The ion-binding site is strictly conserved between the Drosophila dopamine receptor and human SLC6A9. S407G is a non-conservative replacement, where the small and hydrophilic Ser is replaced by a more flexible more hydrophobic glycine without any side chain. This replacement changes the shape and chemical properties of the ion-binding site and, hence, disrupts normal functioning of the transporter.

## Discussion

In this report, we present the first case in the literature of presumptive glycine transporter defect in association with a novel variant in *SLC6A9*. Solute Carrier Family 6 (SLC6), also known as neurotransmitters transporters or Na+/Cl− dependent transporter family, is a group of transporters for neurotransmitters, proteinogenic amino acids, betaine, taurine, and creatine. SLC6 is further subdivided into four subfamilies; monoamine transporters (SLC6A2, SLC6A3, and SLC6A4), GABA transporters (SLC6A1, SLC6A13, and SLC6A11), glycine transporters (SLC6A9, SLC6A5, SLC6A14, and SLC6A7), and neutral amino acid transporter (SLC6A19, SLC6A15, and SLC6A18) (Alexander et al. 2015).

*SLC6A9* encodes GlyT1, which is located in the plasma membrane, predominantly in glial cells and neurons. Its main function is to modulate glycine level in the synapse by transporting glycine from the synaptic cleft into cells. Various experiments showed evidence of crucial role of GlyT1 in the development of basic respiratory pattern and tone as GlyT1 deficiency (*Slc6a9* knockout) in mice causes severe respiratory distress and death shortly after birth (Chalphin and Saha 2010; Gomeza et al. 2003), which faithfully recapitulates our patient’s acute respiratory distress postnatally.

Mayor et al. (1984) case presented with typical NKH symptoms, however, GCS functional study was normal. Furthermore, the glycine transport system was absent in brain autopsy, but no confirmatory mutation analysis was conducted (Mayor et al. 1984).

The presented patient shared similarities with other NKH patients, regarding the age of onset and various clinical features, such as hypotonia, early respiratory distress, myoclonic jerks, and seizures (Table 1), although she also exhibited subtle facial dysmorphic features that have not been described in patients with NKH. More importantly, burst suppression pattern on EEG, which is commonly observed in classical NKH, was not observed in this patient as EEG was reportedly normal. In addition, biochemical profile showed elevation of CSF/plasma glycine ratio (0.07), in contrast to classic NKH patients, where CSF/plasma glycine ratio is usually greater than 0.08. We also note that the proband’s exaggerated startle reflex during the first 6 months of age (hyperekplexia), has previously been reported as a feature in association with *SLC6A5*-related disorders (Arribas-Gonzalez et al. 2015; Dreissen and Tijssen 2012; Michael Davis et al. 1993; Supplisson and Roux 2002), but not in association with NKH, suggesting this feature might be a common finding between GlyT1 and GlyT2 systems. Another possible explanation for the startle reflex could be due to the excitatory effect of glycine on the glutamatergic neurons through *N*-methyl-d-aspartic acid (NMDA) receptors, leading to depolarization of the post-synaptic neurons (Betz et al. 2006), even though such symptoms subsided after 6 months of age.

**Table 1:** Comparison between NKH and glycine transporter 1 defect

Feature	NKH	GlyT1 variant
Age of onset	Neonatal or infantile	Neonatal
Major clinical features	Respiratory distress requiring mechanical ventilation, seizure, hypotonia, microcephaly, spastic quadriplegia, global developmental delay	Respiratory distress requiring mechanical ventilation, microcephaly hypotonia, joint laxity, exaggerated startle response, developmental dysplasia of the hip, global developmental delay
Facial dysmorphic features	None	Broad forehead, esotropia, low set ears, retrognathia, deep prominent philtrum, and sparse eyebrows
Encephalopathy	Present	Absent
Laboratory finding	High CSF, plasma, and urine glycine	High CSF, plasma, and urine glycine
MRI	Delayed myelination, absent corpus callosum, brain atrophy, and dilatation of the ventricles. White matter changes	Atrophy in the caudate nucleus, white matter changes. Normal myelination.
EEG	Burst suppression pattern	Normal

Long-term outcome for mice carrying heterozygous variants in GlyT1 has previously been documented (Gomeza et al. 2003); however, mice carrying homozygous variants expired soon postnatally, rendering it very difficult to predict natural history from structural, developmental, and behavioral aspects. Hypomorphic mutants will potentially clarify the full phenotypic consequences of GlyT1 deficiency in mouse to model what we observed in our human patient and we hope the reported variant can aid in the design of an appropriate knock-in strategy.

In summary, we report the first living human case carrying a homozygous missense variant in *SLC6A9* and the associated phenotype includes: global developmental delay, hypotonia, subtle dysmorphic features, joint laxity, and clubfeet; high plasma and CSF glycine levels with elevated CSF-to-plasma glycine ratio; and brain white matter changes. However, we acknowledge that the disease link we propose in this study is based on a single patient. Further research and additional reports of *SLC6A9* variants are needed to elucidate the full *SLC6A9*-related phenotype and genotype spectrum in humans. In addition, as further delineation of various gene-related phenotypes and identification of more novel genes continue, future reanalysis of whole-exome sequencing data could help clarify if reported dysmorphic features are possibly associated with yet-to-be fully described genes.
